# Multiple oncogenic mutations related to targeted therapy in nasopharyngeal carcinoma

**DOI:** 10.1186/s40880-015-0011-0

**Published:** 2015-04-08

**Authors:** Jian-Wei Zhang, Tao Qin, Shao-Dong Hong, Jing Zhang, Wen-Feng Fang, Yuan-Yuan Zhao, Yun-Peng Yang, Cong Xue, Yan Huang, Hong-Yuan Zhao, Yu-Xiang Ma, Zhi-Huang Hu, Pei-Yu Huang, Li Zhang

**Affiliations:** Sun Yat-sen University Cancer Center; State Key Laboratory of Oncology in South China; Collaborative Innovation Center for Cancer Medicine, Guangzhou, 510060 Guangdong P. R. China; Department of Medical Oncology, Sun Yat-sen University Cancer Center, No. 651 Dongfeng Road East, Guangzhou, 510060 Guangdong P. R. China; Department of Nasopharyngeal Carcinoma, Sun Yat-sen University Cancer Center, Guangzhou, 510060 Guangdong P. R. China

**Keywords:** Nasopharyngeal carcinoma, Oncogenic mutation, SNaPshot

## Abstract

**Introduction:**

An increasing number of targeted drugs have been tested for the treatment of nasopharyngeal carcinoma (NPC). However, targeted therapy-related oncogenic mutations have not been fully evaluated. This study aimed to detect targeted therapy-related oncogenic mutations in NPC and to determine which targeted therapy might be potentially effective in treating NPC.

**Methods:**

By using the SNaPshot assay, a rapid detection method, 19 mutation hotspots in 6 targeted therapy-related oncogenes were examined in 70 NPC patients. The associations between oncogenic mutations and clinicopathologic factors were analyzed.

**Results:**

Among 70 patients, 12 (17.1%) had mutations in 5 oncogenes: 7 (10.0%) had v-kit Hardy-Zuckerman 4 feline sarcoma viral oncogene homolog (*KIT*) mutation, 2 (2.8%) had epidermal growth factor receptor (*EGFR*) mutation, 1 (1.4%) had phosphatidylinositol-4,5-bisphosphate 3-kinase, catalytic subunit alpha (*PIK3CA*) mutation, 1 (1.4%) had Kirsten rat sarcoma viral oncogene homolog (*KRAS*) mutation, and 1 (1.4%) had simultaneous *EGFR* and v-Raf murine sarcoma viral oncogene homolog B1 (*BRAF*) mutations. No significant differences were observed between oncogenic mutations and clinicopathologic characteristics. Additionally, these oncogenic mutations were not associated with tumor recurrence and metastasis.

**Conclusions:**

Oncogenic mutations are present in NPC patients. The efficacy of targeted drugs on patients with the related oncogenic mutations requires further validation.

## Background

Nasopharyngeal carcinoma (NPC) is characterized by a unique geographic distribution [1]. The incidence of NPC in South China is one of the highest worldwide (20 to 30 per 100,000) [2]. Radiotherapy with or without chemotherapy is the standard of care for non-metastatic disease [3]. With the development of radiotherapy techniques, the prognosis of early-stage NPC has been obviously improved, as indicated by a 5-year local control rate of 95% and a 5-year disease-free survival rate of 77% [4]. Distant metastasis, which occurred in approximately 20% of patients with locally advanced disease, was the main reason for treatment failure [4]. For patients with metastatic disease, the median overall survival (OS) was less than 2 years.

Currently, with the rapid development of molecular medicine, an increasing number of targeted drugs have been developed and used in cancer treatment, thereby providing new options for the management of NPC. Several clinical trials testing targeted therapy in NPC demonstrated the promising efficacy of targeted drugs such as cetuximab [5], bevacizumab [6], and sorafenib [7]. However, none of these targeted drugs have been approved by the Food and Drug Administration (FDA) for use in NPC due to the limited improvement on survival. In fact, not all of the patients could benefit from targeted therapy. The sensitivity of targeted drugs is related to the genetic makeup of individual tumors; therefore, the identification of somatic mutations is increasingly important in the clinical management of cancer [8].

Different types of targeted drugs have been tested in clinical trials for NPC, including monoclonal antibodies against epidermal growth factor receptor (EGFR) and vascular endothelial growth factor (VEGF) as well as small-molecule tyrosine kinase inhibitors (TKIs) [5-7]. These drugs had different mechanisms of inhibiting molecular signaling pathways. Monoclonal antibodies against EGFR, such as cetuximab and nimotuzumab, bind to EGFR to block its downstream signaling pathway. Additionally, the monoclonal antibody against VEGF had anti-angiogenic effects. Among small-molecule TKIs, such as sorafenib, famitinib, and dasatinib, most were multi-targeted TKIs. However, until now, few studies have focused on the roles of oncogenic mutations in NPC, and the results are controversial. No specific oncogenic target has yet been identified in NPC for targeted therapy [9].

Here, we aimed to evaluate the prevalence of targeted therapy-related oncogenic mutations in NPC using the SNaPshot assay and to identify potentially effective targets in NPC. We mainly focused on the EGFR signaling pathway components, including *EGFR,* phosphatidylinositol-4,5-bisphosphate 3-kinase, catalytic subunit alpha (*PIK3CA*), Kirsten rat sarcoma viral oncogene homolog (*KRAS*), and v-Raf murine sarcoma viral oncogene homolog B1 (*BRAF*). In addition, the v-kit Hardy-Zuckerman 4 feline sarcoma viral oncogene homolog (*KIT*) gene was also analyzed. All of these factors are common hotspots for mutations in cancer*.*

## Methods

### Patient selection

Consecutive patients who were pathologically diagnosed with NPC between April 2012 and December 2012 at the Sun Yat-sen University Cancer Center (Guangzhou, China) for whom fresh-frozen tissue samples were available were enrolled. The clinicopathologic information of all patients was collected, including sex, age, tumor stage, pathologic type, and treatment regimen. Tumor stage was classified according to the Union for International Cancer Control (UICC) 2010 NPC TNM staging system. Fresh nasopharyngeal tissue samples were obtained from all patients. Informed consent forms outlining tissue collection and clinical information analyses were signed by all patients. The protocol was approved by the institutional ethics committee of the Sun Yat-sen University Cancer Center.

### DNA extraction

Fresh frozen tissues confirmed to be NPC by hematoxylin and eosin staining were obtained and stored in microtubes at −80°C. DNA was extracted by using the QIAamp DNA extraction kit (QIAGEN, Shanghai, China) according to the manufacturer’s protocol. Briefly, these tissues were transferred to 1.5-mL Eppendorf tubes, treated with animal tissue lysis buffer, and incubated with proteinase K at 56°C overnight. After tissue lysis, 200 μL alkaline lysis buffer and 200 μL ethanol were added. The lysate was then transferred to the QIAamp MinElute column, 20 μL elution buffer was added, and the DNA was collected.

### SNaPshot assay

Multiple oncogenic mutations were detected with the SNaPshot multiplex kit (QIAGEN, Shanghai, China), which involves multiplex polymerase chain reaction (PCR), multiplexed single-base primer extension, and capillary electrophoresis. The experiment was performed according to the instructions of the kit. Five oncogenes, *EGFR*, *PIK3CA*, *KRAS*, *BRAF*, and *KIT*, were detected, which were mainly involved in the EGFR signaling pathway. The SNaPshot assay is more flexible and slightly more sensitive than direct sequencing [10,11].

### Statistical analysis

The associations between oncogenic mutations and clinicopathologic parameters were assessed with Fisher’s exact test or Student’s *t* test. A *P* value of less than 0.05 was considered significant. SPSS software 16.0 (SPSS Inc., Chicago, IL, USA) was used in the statistical analysis.

## Results

### Patient characteristics

A total of 70 patients were enrolled for the analysis. Among the 70 patients, 46 were men and 24 were women, with a median age of 46 years (range, 24 to 73 years). According to the WHO histological classification, all patients were diagnosed with non-keratinizing undifferentiated carcinoma. The median follow-up duration was 25.8 months, with January 2014 as the last follow-up date. Only 11 patients had tumor recurrence or metastasis. The baseline characteristics of all patients are shown in Table 1.

**Table 1:** **Clinical characteristics of 70 patients with nasopharyngeal carcinoma (NPC)**

**Characteristic**	**Number of patients (%)**
**Sex**	
Man	46 (65.7)
Women	24 (34.3)
**Smoking status**	
Smoker	29 (41.4)
Non-smoker	41 (49.2)
**Clinical stage**	
I	2 (2.9)
II	14 (20.0)
III	39 (55.7)
IV	15 (21.4)
**EBV copy number**	
>1,000	59 (84.3)
<1,000	11 (15.7)
**Therapy**	
No treatment	1 (1.4)
Radiotherapy	16 (22.9)
Chemoradiotherapy	53 (75.7)
**Recurrence or metastasis**	
Yes	11 (15.7)
No	59 (84.3)

EBV, Epstein-Barr virus.

### Oncogenic mutation profiles

**Figure 1:**
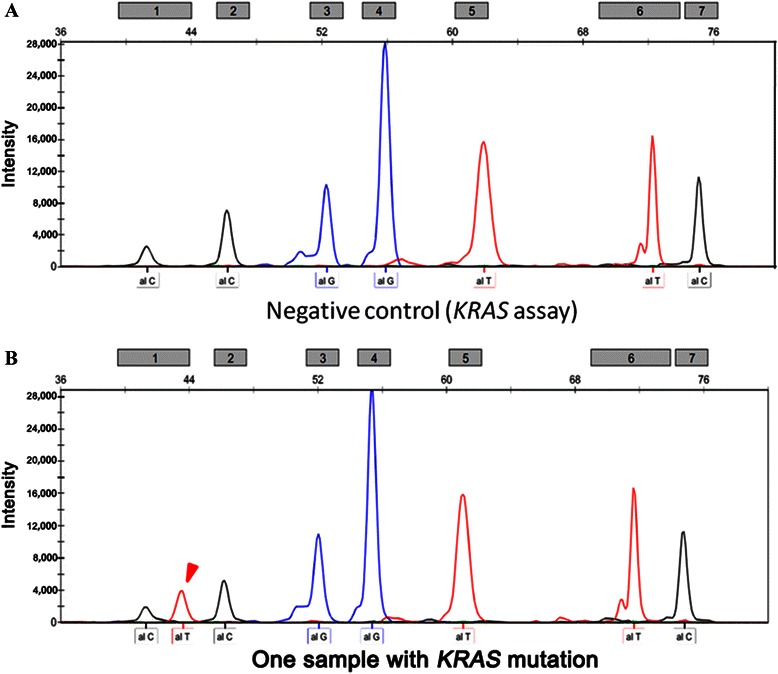
**Representative images showing the** ***KRAS*** **mutation detected by the SNaPshot assay. A**, a negative control sample with no mutation peak; this sample was from a confirmed patient with no mutation. **B**, a positive sample with a typical *KRAS* mutation peak (red arrow head).

**Figure 2:**
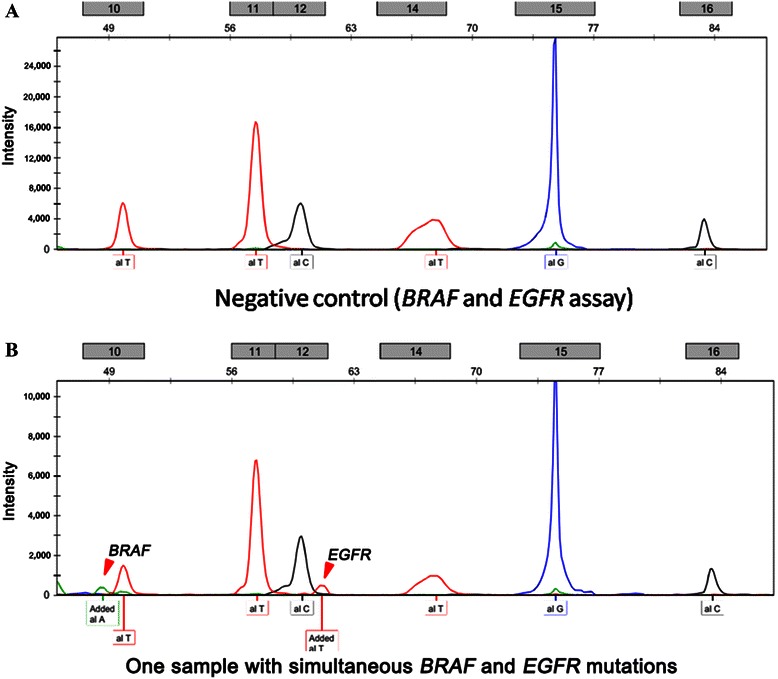
**Representative images showing simultaneous** ***BRAF*** **and** ***EGFR*** **mutations detected by the SNaPshot assay. A**, a negative control sample with no mutation peak; this sample was from a confirmed patient with no mutations. **B**, a positive sample with typical *BRAF* and *EGFR* mutation peaks (red arrow heads).

Nineteen mutation hotspots of 5 targeted therapy-related oncogenes were examined in 70 NPC patients. Among the 70 patients, 12 (17.1%) had mutations in 5 oncogenes: 7 (10.0%) had *KIT* (M541L) mutation, 2 (2.8%) had *EGFR* (T790M) mutation, 1 (1.4%) had *PIK3CA* (H1047R) mutation, 1 (1.4%) had *KRAS* (G13D) mutation, and 1 (1.4%) had simultaneous *EGFR* and *BRAF* (V600E) mutations (Figures 1 and 2).

### Associations between oncogenic mutations and clinicopathologic characteristics

Between the mutation and wild-type groups, no significant differences were observed for the clinicopathologic factors, including age, sex, clinical stage, EBV copy number, and recurrence/metastasis (Table 2). Only 11 patients had disease recurrence and metastasis; hence, survival analysis was not performed in this study.

**Table 2:** **Associations between oncogenic mutations and clinical characteristics of NPC**

**Parameter**	**Mutation group**	**Wild-type group**	***P***
**Total**	12	58	
**Age (years)**			0.342
>46	4	31	
≤46	8	27	
**Sex**			0.526
Man	9	37	
Women	3	21	
**Clinical stage**			1.00
I + II	3	13	
III + IV	9	45	
**EBV copy number**			0.092
>1,000	11	38	
<1,000	1	20	
**Recurrence or metastasis**			0.675
Yes	1	10	
No	11	48	

The Fisher’s exact test was performed in this analysis.

## Discussion

In this study, with the rapid detection method, we found that *KIT* mutation was the most frequent in NPC followed by *EGFR* mutation, whereas *PI3KCA*, *KRAS*, and *BRAF* mutations were less frequent, which was consistent with the results of a previous study [12].

No obvious association was found between oncogenic mutations and clinicopathologic factors. Although another retrospective study showed that NPC patients who had recurrence or developed metastases had high mutation frequencies due to the relatively short follow-up duration, our study did not find the same result.

KIT is a transmembrane tyrosine kinase receptor encoded by the proto-oncogene *C-KIT*. The activation of KIT is associated with tumor proliferation, differentiation, adhesion, and apoptosis [13]. KIT was reported to be expressed in 33%-88% of NPCs [14-16]. Additionally, *KIT* mutation was also reported in our previous study [17]. It is well known that the *KIT* oncogene inhibitor imatinib has been widely used in clinical practice and has been approved for use in melanoma, gastrointestinal stromal tumor (GIST), and chronic myelogenous leukemia (CML) patients with *KIT* mutations [18,19]. In our study, a *KIT* mutation in exon 10 (M541L), which is different than the functional mutations in exon 9 and exon 11 [20], occurred in 10% of NPC patients. However, the *KIT* (M541L) mutation was considered a gene polymorphism in CML. A myeloid cell line with the *KIT* (M541L) mutation was sensitive to imatinib in preclinical studies [21,22]. In addition, in previous experiments, imatinib could inhibit the proliferation of NPC cell lines (CEN-1, CNE2, Hone-1 C-666, SUNE-1, and 5-8 F) in a dose-dependent manner [17]. Thus, whether imatinib could be used in NPC patients with *KIT* mutations remains unknown, and further clinical trials are necessary to validate the efficacy of imatinib for treating NPC patients with *KIT* mutations.

EGFR is also a transmembrane tyrosine kinase receptor. EGFR overexpression in NPC is quite common and has been reported with a prevalence as high as 80% in primary NPC biopsies [23-25]. It has been proven that EGFR overexpression was indicative of poor prognosis and was associated with a low local control rate and short survival [26,27]. Thus, *EGFR* is considered an important target in NPC. However, *EGFR* mutations are reported to have an extremely low prevalence (0-1%) in NPC [28,29]. In our study, *EGFR* mutations were detected in 3 (4.3%) patients, all of whom had the T790M mutation in exon 20. In a phase II clinical trial of gefitinib for treating NPC, there was no response. The median progression-free survival (PFS) and OS were 4 and 16 months, respectively [30]. This result could be explained by the low *EGFR* mutation rate in NPC. In addition, patients with the T790M mutation showed primary resistance to gefitinib [31]. However, a new generation of EGFR TKIs, including AZD9291 and CO-1686, that target T790M has been recently developed [32,33]. Perhaps a small subset of NPC patients could benefit from these new EGFR TKIs.

Phosphatidylinositol 3 kinase (PI3K) is involved in diverse cellular functions, including cell proliferation, differentiation, motility, and survival. The PI3K-AKT pathway plays an important role in different tumor types. Activation of the PI3K pathway is significant in the development of NPC [34,35]. The *PI3KCA* mutation rate was low. In the present study, only 1 patient (1.4%) had *PI3KCA* (H1047Y) mutation. The treatment of NPC cells with the PI3K inhibitor LY294002 led to the inhibition of AKT activation [36]. In addition, another inhibitor, MK-2206, which targets AKT, abrogates the AKT signaling [37]. These drugs are currently being evaluated in clinical trials. The PI3K-AKT pathway might be a new target in NPC [29].

The RAS/RAF/ERK pathway, which also plays an important role in tumor development, is downstream of EGFR [38,39]. Previous studies detected no *KRAS* mutations in NPC specimens or cell lines [29,40]. In the present study, *KRAS* mutations were detected in only 1 patient. Mutations of the Ras signaling pathway might lead to cetuximab resistance in NPC [41]. Furthermore, the *BRAF* (V600E) mutation was detected in only 1 patient. *BRAF* mutations were more common in melanoma, and a BRAF inhibitor has been approved for the treatment of melanoma. Although the mutation rate is not high, *BRAF* is also a candidate biomarker for targeted therapy.

This study has several limitations. First, only 5 oncogenes related to the available targeted drugs were selected in our study. Second, the follow-up duration was short, and survival analysis was not conducted. Third, compared with deep sequencing, the SNaPshot method provides high-throughput data and is less expensive; however, it was not adequate to detect all genes in NPC. Therefore, our team is preparing to conduct whole-genome sequencing for NPC, which might provide more molecular information for NPC in the near future.

## Conclusions

In our study, we found that 10% of patients with NPC had *KIT* mutations that were not at the commonly mutated sites. Additionally, other hotspot oncogenic mutations were infrequent in NPC patients from South China. The discovery of new therapeutic targets is underway.
